# Molecular identification and functional characterization of the cathepsin B gene (*Ab-cb-1*) in the plant parasitic nematode *Aphelenchoides besseyi*

**DOI:** 10.1371/journal.pone.0199935

**Published:** 2018-06-29

**Authors:** Hong-Le Wang, Xi Cheng, Shan-Wen Ding, Dong-Wei Wang, Chun Chen, Chun-Ling Xu, Hui Xie

**Affiliations:** 1 Laboratory of Plant Nematology and Research Center of Nematodes of Plant Quarantine, Department of Plant Pathology / Guangdong Province Key Laboratory of Microbial Signals and Disease Control, College of Agriculture, South China Agricultural University, Guangzhou, People’s Republic of China; 2 Key Laboratory of Biopesticide and Chemical Biology, Ministry of Education, College of Plant Protection, Fujian Agriculture and Forestry University, Fuzhou, People’s Republic of China; Stony Brook University, UNITED STATES

## Abstract

The rice white tip nematode, *Aphelenchoides besseyi*, is widely distributed in rice planting areas worldwide and causes serious economic losses. Cathepsin genes have been demonstrated to have importance in studying the reproduction, development, pathogenicity, and control methods of plant nematodes. In this paper, a novel cathepsin B gene, *Ab-cb-1*, was found and cloned. The *Ab-cb-1* gene was 1347 bp in length and encodes 369 amino acids. The *Ab-*CB-1 protein contains characteristic occluding loops but no signal peptide. A homology analysis showed that *Ab-*CB-1 had the highest identity value (64%) to the known amino acid sequence of cathepsin B-like cysteine protease 6 from *Toxocara canis*. When *Ab-cb-1* was expressed in a prokaryotic system, the protein massed approximately 45 kDa and could decompose carrot callus. *Ab-cb-1* mRNA was localized in the nematode intestine. The relative expression level of *Ab-cb-1* in the *A*. *besseyi Ab*-S24 population, which had high reproductivity, was approximately 6.9 times that in the *Ab-*N10 population, which had low reproductivity, and the difference was significant (*p*<0.05). The *Ab-cb-1* expression level was highest in females; the expression levels in males, juveniles and eggs were 30%, 12.2% and 5% of that in females, respectively, and the differences were significant among all four stages (*p*<0.05). Nematodes of the *Ab*-S24 population were treated with *Ab-cb-1* dsRNA for 12 h, 24 h, 36 h and 48 h, and their reproduction decreased with increasing time. These results demonstrated that *Ab*-CB-1 was a digestive enzyme with hydrolytic protease properties and that *Ab-cb-1* played an important role in the reproduction of *A*. *besseyi*.

## Background

*Aphelenchoides besseyi* is a migratory plant parasitic nematode with more than 200 host plants in over 35 genera. The main hosts are rice (*Oryza sativa*) and strawberry (*Fragaria ananassa*) [1]. *A*. *besseyi* is distributed over almost all the world’s rice planting areas, and it is one of the major rice seed-borne diseases that cause serious economic losses [2, 3, 4].

Cathepsins belong to the cysteine protease family and are commonly found in parasites as digestive enzymes. Cathepsins have hydrolytic protease properties [5, 6] and play important physiological and biochemical roles. In recent years, studies have shown that cathepsin genes are essential in the physiological and biochemical processes of parasites and insects, such as hatching, reproduction, development, infection, pathogenicity and immune evasion [7]. Several cathepsins including cathepsin L, cathepsin S, and cathepsin B have been demonstrated to have importance in plant parasitic nematodes, and thus far, cathepsin L has been the subject of greatest interest [8, 9]. So far, several cathepsin L genes have been successfully cloned from a variety of plant nematodes including *Heterodera avenae* (ACJ13100), *H*. *glycines* (Y09498), *H*. *schachtii* (ACJ13098), *Globodera virginiae* (ACJ13094), *G*. *mexicana* (ACJ13096) *Meloidogyne incognita* (CAD89795), *Rotylenchulus reniformis* (AAY45870), *Bursaphelenchus xylophilus* (ACH56225), and *Ditylenchus destructor* (GQ180107). *Mi-cpl-1*, a cathepsin L gene from *M*. *incognita*, has been shown to be correlated with parasite success and to be essential in the interaction between nematodes and plants [10], so this gene could be further developed for root-knot nematode control. Cathepsin S genes (*cps*) have also been successfully cloned from plant parasite nematodes including *H*. *glycines* [9], *H*. *avenae* [11] and *Radopholus similis* (EU659125) [12]. The *cps* gene in *R*. *similis* has been confirmed to be related to reproduction, parasitism and pathogenicity of the nematode [13]. However, cathepsin B genes (*cb*) in plant parasitic nematodes are seldom reported and have been cloned in only *B*. *xylophilus* (GU130153) and *R*. *similis* (GU360972). The *Rs-cb-1* gene found in *R*. *similis* has been reported as a key gene that could affect its development and pathogenicity [14, 15].

In view of the important role and application of the cathepsin gene in the reproduction, development, parasitism and control of nematodes, a novel cathepsin gene was screened from the cDNA library of *A*. *besseyi* in this study and successfully cloned. Then, its biological characteristics and functions were identified for the first time.

## Methods

### Nematode and cultivation

The *A*. *besseyi* populations used in this study were the *Ab*-S24 population collected from strawberry (*F*. *ananassa*) in Longgang District, Shenzhen, Guangdong, China, and the *Ab*-N10 population collected from rice (*O*. *sativa*) in Luhe Town, Nanjing, Jiangsu Province, China. Nematodes were isolated, identified, preserved and cultured by the Laboratory of Plant Nematology, South China Agricultural University. The reproductivity was found to be higher in *Ab*-S24 than in *Ab*-N10 [16]. The preservation and cultivation of the nematodes were carried out using the method described by Cheng et al. [1].

### Cloning of the full-length *Ab-cb*-1 gene from *A*. *besseyi*

Total RNA of approximately 20,000 mixed-stage nematodes of the *Ab*-S24 population was extracted using the Invitrogen TRIzol^®^ Reagent kit (Invitrogen, Carlsbad, CA, USA) and reverse transcribed into cDNA using RQ1 RNase-Free DNase (Promega, Madison, WI, USA). Both 3' RACE primers (CB-F1, CB-F2) and 5' RACE (CB-R1, CB-R2) (Table 1) primers were designed for cDNA amplification according to the conserved expressed sequence tag (EST) sequences of *A*. *besseyi* similar to the cathepsin B gene found in our previous study. The amplified products were purified and ligated with pMD 18-T vector (Takara, Japan) to obtain recombinant plasmid. Recombinant plasmid was transformed into *Escherichia coli* JM109 competent cells, and then positive clones were selected for sequencing (BGI Company). According to the sequencing results, primers QCCF and QCCR (Table 1) were designed for the full-length amplification of the *Ab-cb-1* gene from *A*. *besseyi*.

**Table 1:** Primers used in this study.

Primers	Sequences	Primer use
CB-F1	5′-TGCGGATTTGGTTGC-3′	3’ RACE
CB-F2	5′-CAAACCATGCCCAAAGGAACTATATC-3′	3’ RACE
CB-R1	5′-TTCCTTTGGGCATGGTTTGAAATGAG-3′	5’ RACE
CB-R2	5′-CTGGGAAAGTGTACGGTTG-3′	5’ RACE
QCCF	5′-AAATGTTGGCGAAGTTAAGTGTAGC-3′	*Ab-cb-1*
QCCR	5′-CAATCGAGCGAAATGTAAAATAAAA-3′	*Ab-cb-1*
CBfBamHI	5′-CGGGATCCATGAAGACAAAAAACAATGATTTG-3′	*Ab-cb-1* plasmid
CBrXhoI	5′-CCGCTCGAGTTAGAAAATATCATAGGAACTAGCC-3′	*Ab-cb-1* plasmid
qPCRC-F	5′-TGAATGTTAGAAACCCAATCAAAG-3′	qPCR
qPCRC-R	5′-CACTACGACACATTGAACCCCA-3′	qPCR
18sF	5′-CTCGTGGTGGCTGGTATGCTG-3′	qPCR
18sR	5′-GTTTCCCGTGTTGAGTCAAATTAAG-3′	qPCR
CB-IN-T7S1	5′-TAATACGACTCACTATAGGGATCTTGTGGCTCCTGTTGGTCAT-3′	RNA probe
CB-IN-A1	5′-TCCGTTTGAGTGAATGCAGATGC-3′	RNA probe
CB-IN-T7A1	5′-TAATACGACTCACTATAGGGTCCGTTTGAGTGAATGCAGATGC-3′	RNA probe
CB-IN-S1	5′-ATCTTGTGGCTCCTGTTGGTCAT-3′	RNA probe
CBRiF	5′-CCGCAACATCAAACAACAAATTCGC-3′	RNA probe
CBRiT7R	5′-TAATACGACTCACTATAGGGCGGCTCCAAATGACCAACAGG-3′	dsRNA template
CBRiT7F	5′-TAATACGACTCACTATAGGGCCGCAACATCAAACAACAAATTCGC-3′	dsRNA template
CBRiR	5′-CGGCTCCAAATGACCAACAGG-3′	
G-T7A	5′-GGATCCTAATACGACTCACTATAGGGCGATGCGGTTCACCAGGGTGTCG-3′	dsRNA template
G-S	5′-CACAAGTTCAGCGTGTCCGGCG-3′	dsRNA template
G-T7S	5′-GGATCCTAATACGACTCACTATAGGGCACAAGTTCAGCGTGTCCGGCG-3′	dsRNA template
G-A	5′-CGATGCGGTTCACCAGGGTGTCG-3′	dsRNA template

### Sequence analysis, alignment and phylogenetic analysis of *Ab*-CB-1

Sequence homology alignments were performed using BLASTN and BLASTX (http://blast.ncbi.nlm.nih.gov/Blast.cgi). Protein bioinformatic analysis was performed using Protein Machine software (http://www.expasy.ch/tools/), including predictions of protein transmembrane region, amino acid sequence, isoelectric point analysis, molecular weight and hydrophobicity analysis. Predictions of signal peptide and cleavage site were performed at http://www.cbs.dtu.dk/services/SignalP/. The phylogenetic tree was constructed using the neighbor-joining method [17] with the program MEGA (Molecular Evolutionary Genetics Analysis, USA) based on *Ab*-CB-1 and other 26 cathepsin B amino acid sequences from 17 species of representative nematodes in the NCBI database.

### Prokaryotic expression and purification of recombinant *Ab*-CB-1

The full-length *Ab-cb-1* gene was amplified from the plasmid with primers CBfBamHI and CBrXhoI (Table 1). The amplified product was purified by digestion with BamHI and XhoI (Takara DNA Fragment Purification Kit Ver 2.0), ligated to prokaryotic expression vector pET-28 (+) (Novagen, Madison, WI, USA), and subsequently introduced to *E*. *coli* JM109 for sequence confirmation. The recombinant plasmid thus obtained was introduced into *E*. *coli* BL21 (DE3) competent cells for prokaryotic expression using the method described by Cheng et al [1]. The recombinant fusion *Ab*-CB-1 protein with His-tag at the N-terminus was purified by affinity chromatography using Ni Sepharose High Performanc (GE Healthcare, Sweden) according to the manufacturer’s instructions. The purity of purified recombinant protein was confirmed by SDS-PAGE. Concentration of purified *Ab*-CB-1 was determined using Easy ⅡProtein Quantitative Kit (BCA) (Transgene, China).

### Decomposition of carrot callus by recombinant *Ab*-CB-1

*E*. *coli* BL21 cells containing *Ab*-CB-1 constructs were collected from 10 mL bacterial suspension and induced to express the *Ab-cb-1* gene by centrifugation (10,000 rpm, 1 min). These bacterial cells were broken ultrasonically for 30 min after mixing with PBS buffer, and the supernatant was obtained as a primary enzyme extract. Aliquots of 100 μL of each primary enzyme extract (from induced and non-induced *E*. *coli* cells containing *Ab*-CB-1 constructs), purified *Ab*-CB-1 (1.0 mg/ml) and sterile water were inoculated into carrot callus under sterile conditions, and then the callus was cultured at 25°C to observe changes in the carrot tissue.

### Expression levels of *Ab-cb*-1 in *Ab*-S24 and *Ab-*N10

Total RNA was extracted from approximately 20,000 mixed-stage nematodes of *Ab*-S24 and *Ab*-N10 populations using the Invitrogen TRIzol® Reagent kit. RNA from different development stages of the *Ab*-S24 population was extracted from 500 each of females, males, juveniles and eggs using a MicroElute Total RNA kit (Omega, USA). The extracted RNA was reverse transcribed into cDNA using the RQ1 RNase-Free DNase (Promega) reverse transcription kit as described above. The expression levels of *Ab-cb-1* in the *Ab*-S24 and *Ab*-N10 populations and in four different development stages of the *Ab*-S24 population were detected on a CFX-96 (Bio-Rad) qPCR machine with cDNA as a template using a SYBR Green Real-time PCR Master Mix Plus kit (Toyobo, Japan). Specific primers qPCRC-F and qPCRC-R (Table 1) were designed to detect *Ab-cb-1* expression. A 140 bp sequence of 18S rRNA (AY508035) was amplified as a reference gene using the primers 18sF and 18sR (Table 1). The qPCR data were analyzed using the CFX Manager software provided by Bio-Rad. All experiments were performed in three biological replicates and each in three replicates.

### *In situ* hybridization of *Ab-cb*-1

*In situ* hybridization was performed as described previously [1,18]. Approximately 10,000 nematodes of mixed stages of the *Ab*-S24 population were collected and concentrated into 30–50 μl. The nematodes were fixed in 3% paraformaldehyde for 18 h at 5°C and then at 22°C for 4 h. DIG-labeled sense and antisense RNA probes (Roche, Germany) were synthesized using sense primers (CB-IN-T7S1, CB-IN-A1) and antisense primers (RB-IN-TA1, CB-IN-S1) (Table 1) based on the full-length cDNA of *Ab-cb-1*. After adding the obtained DIG-labeled RNA probes, the hybridization solution containing nematodes was rotated for 12 h at 47°C. The results were examined and photographed by differential interference microscopy.

### dsRNA synthesis and RNAi efficiency of *Ab-cb-*1

The RNAi knockdown of the *Ab-cb-1* gene was performed by soaking the nematodes with double-stranded RNA (dsRNA) of *Ab-cb-1* synthesized by in vitro transcription. Two primer pairs, CBRiF/CBRiT7R and CBRiT7F/CBRiR (Table 1), were designed to amplify the sense and antisense single-stranded RNA (ssRNA) products, respectively. *Ab-cb-1* dsRNA was synthesized according to the instructions of the Script MaxTM Thermo T7 Transcription kit (Toyobo). The obtained dsRNA synthesis product was purified using the described method, then examined for integrity by electrophoresis, detected for concentration and quality by Nanodrop spectrophotometer, analyzed by 1.2% agarose gel electrophoresis, and finally stored at -80°C for later use. The non-endogenous control (green fluorescent protein gene, *gfp*) dsRNA (125 bp) was generated with the specific primers G-T7S, G-A and G-S (Table 1) [19]. Five hundred mixed-stage nematodes of the *Ab-S24* population were separated from carrot callus and collected in a diethyl pyrocarbonate (DEPC)-treated centrifuge tube. A 50 μl aliquot of *Ab-cb-1* dsRNA (2 μg/μL) was added into the tube to soak the nematodes at 25°C. The nematodes were soaked for 12 h, 24 h, 36 h, and 48 h, respectively. Non-endogenous *gfp* dsRNA solution 50 ml (2 μg/μL) was used as a control. In total, eight treatments were performed in triplicate. The RNA of the nematodes treated by soaking was extracted after washing the nematodes three times with DEPC-treated water. RNAi efficiency was examined by determining the *Ab-cb-1* expression level by qPCR.

### Effect of *Ab-cb-*1 RNAi on nematode reproduction

Females of the *Ab*-S24 population were treated with *Ab-cb-1* dsRNA for 12 h, 24 h, 36 h and 48 h and treated with *gfp* dsRNA as control. A total of 30 female nematodes were selected from each treatment and inoculated on carrot callus, and each treatment was repeated five times. Carrot callus dishes inoculated with nematodes were incubated at 25°C in the dark for 35 days, and then nematodes on carrot callus were separated and counted.

### Statistical analysis

All data in this study were subjected to analysis of variance (ANOVA), and multiple comparisons of means were conducted by Duncan’s Multiple Range Test at *p* = 0.05 using SAS (Release 8.01).

## Results

### Cloning of the full-length *Ab-cb-*1 gene from *A*. *besseyi*

A 1347 bp full-length cDNA sequence from *A*. *besseyi* was amplified using the specific primers QCCF and QCCR (Table 1) and confirmed by sequencing. The cDNA sequence was named *Ab-cb-1* and included a 1110 bp open reading frame (ORF) found with NCBI ORF finder, encoding 369 amino acids (GenBank accession number JQ686691). *Ab-cb-1* began with an ATG initiation codon after 129 bp of upstream 5' untranslated region and ended with a TAA stop codon before 108 bp of downstream 3' untranslated region.

### Sequence analysis, alignment and phylogenetic analysis of *Ab*-CB-1

The *Ab-*CB-1 protein sequence (S1 Fig) translated from the *Ab-cb-1* gene sequence encoded 369 amino acids with a theoretical molecular mass of 41.8718 kDa. Sequence homology alignment analysis showed that *Ab-*CB-1 was a cathepsin B gene in the cysteine protease family. The results of protein bioinformatic analysis showed that *Ab-*CB-1 had the typical characteristics of the cysteine protease family: a cysteine protease catalytic triad of residues containing the sites cysteine (Cys, 122), histidine (His, 293) and asparagine (Asn, 313); two occluding loops (between residues 116 and 127 and between residues 291 and 301); glutamine oxyanion hole (116); predicted cleavage point between the mature and pro-domains (93); hemoglobinase motif (residues between 308 and 321); and protease S_2_ pocket (residues between 335 and 339).

The sequence homology alignment showed that the highest homology with the *Ab*-CB-1 sequence was found in cathepsin B-like cysteine protease 6 from *Toxocara canis* (GenBank accession: KHN84702.1, similarity 73%, identity 64%, E value: 3e-156), followed by two cathepsin B-like cysteine proteases from *Ascaris suum* (GenBank accessions: AAB40605.1 and ERG84961.1, similarity 73%, identity 62%, E value 2e-154) and hypothetical protein Y032_0897g2931 from *Ancylostoma ceylanicum* (GenBank accession: EYC36434.1, similarity 75%, identity 60%, E value 5e-153).

**Fig 1:**
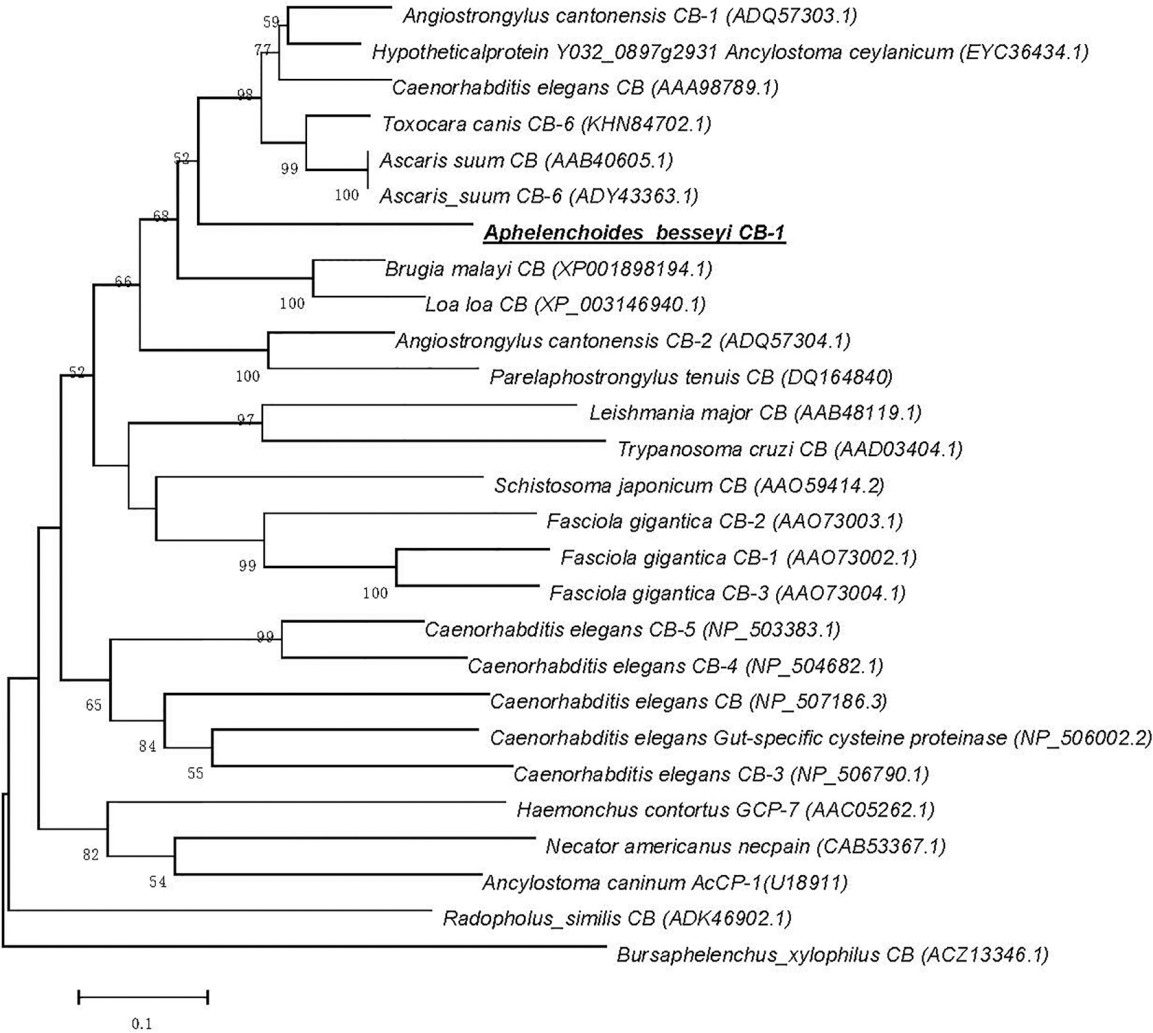
Phylogram constructed on the basis of amino acid sequences depicting evolutionary relationships among the cathepsin B genes of 16 parasitic species. *Aphelenchoides besseyi* CB-1 is highlighted by the underline; accession numbers of the sequences are shown in brackets; distances on the *X*-axis correspond to the degree of homology between sequences; distances on the *Y*-axis are arbitrary.

The phylogenetic tree was constructed based on *Ab*-CB-1 and other 26 cathepsin B amino acid sequences from 17 species of representative parasites in the NCBI database (Fig 1). The result showed that *Ab*-CB-1 had the closest overall relationship with cathepsin B-like cysteine protease 6 from *Toxocara canis*, which was consistent with the results of the alignment analysis obtained by blastx.

### Prokaryotic expression of recombinant *Ab*-CB-1 and purification

**Fig 2:**
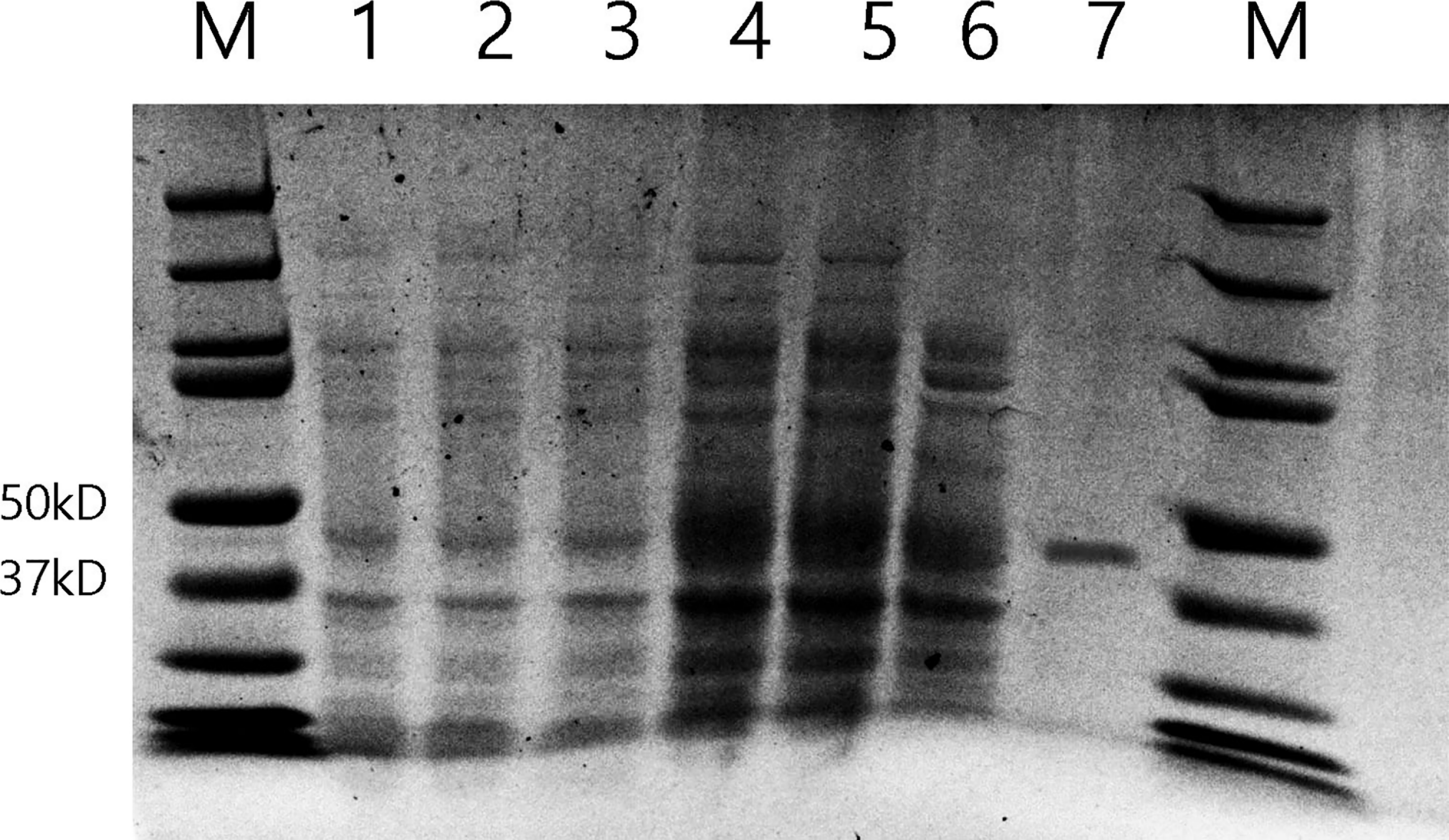
Sodium dodecyl sulfate polyacrylamide gel of lysate of *Escherichia coli* cells expressing *Ab*-CB-1 from the plasmid vector pET-28a (+). M: PageRuler Prestained Protein Ladder (Thermo, USA); 1, 2: Protein extracts from induced *E*. *coli* cells harboring the empty pET-28a (+) vector by 0.5 and 1.0 mM IPTG; 3: Protein extract from non-induced *E*. *coli* cells containing the *Ab*-CB-1 construct; 4,5: Protein extracts from *E*. *coli* cells containing the *Ab*-CB-1 construct and induced by 0.5 and 1.0 mM IPTG; 6: Supernatant of cell lysate from *E*. *coli* cells containing the *Ab*-CB-1 construct; 7 Purified recombinant *Ab*-CB-1.

The expression of pET-28a (+)-*Ab*-CB-1 was induced by isopropyl β-D-1-thiogalactopyranoside (IPTG), and the recombinant protein extract was examined by SDS-PAGE. Compared with non-induced *E*. *coli* containing the *Ab*-CB-1 construct and induced *E*. *coli* harboring the empty pET-28a (+) vector, only the IPTG-induced recombinant had an obvious band at 45 kDa (Fig 2). Prokaryotic expression of recombinant *Ab*-CB-1 was confirmed, and protein extract of *Ab*-CB-1 was obtained.

### Decomposition of carrot callus by purified recombinant *Ab*-CB-1

**Fig 3:**
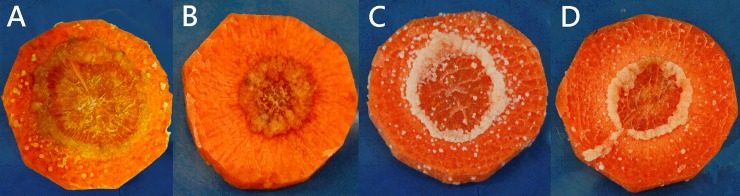
Inoculation of carrot callus with prokaryotically expressed products of *Ab-cb-1*. A: carrot callus inoculated with the purified protein *Ab*-CB-1 in 1mg/ml; B: carrot callus inoculated with the primary enzyme extract from induced *E*. *coli* cells containing the *Ab*-CB-1 construct; C: carrot callus inoculated with distilled water; D: carrot callus inoculated with the primary enzyme extract from non-induced *E*. *coli* cells containing the *Ab*-CB-1 construct.

After inoculation with the purified protein *Ab*-CB-1, and the primary enzyme extract from induced *E*. *coli* cells containing the *Ab*-CB-1 construct, carrot callus changed color to yellow-brown and decomposed visibly (Fig 3A and 3B). In the treatments with sterile water (Fig 3C) and the primary enzyme extract from non-induced *E*. *coli* cells containing the *Ab*-CB-1 construct (Fig 3D), carrot callus remained white in color and was not visibly decomposed (Fig 3D). These results showed that the primary enzyme extract from prokaryotic expression of recombinant *Ab*-CB-1 could decompose carrot callus.

### *Ab-cb*-1 expression in two populations and four developmental stages of *A*. *besseyi*

**Fig 4:**
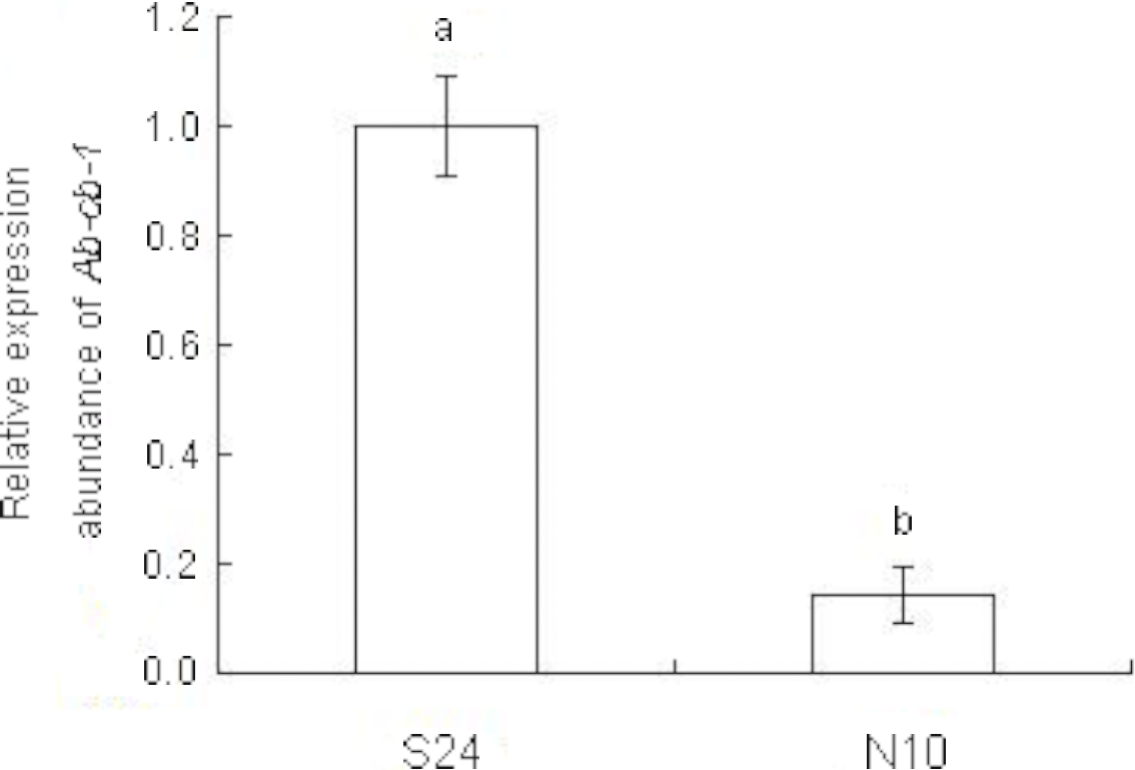
Expression levels of *Ab-cb-1* in two populations of *Aphelenchoides besseyi*. S24, N10: populations of *A*. *besseyi* collected from *Fragaria ananassa* and *Oryza sativa*, respectively; bars indicate standard errors of the mean (n = 3), and different letters indicate significant differences (*p*<0.05) between treatments.

**Fig 5:**
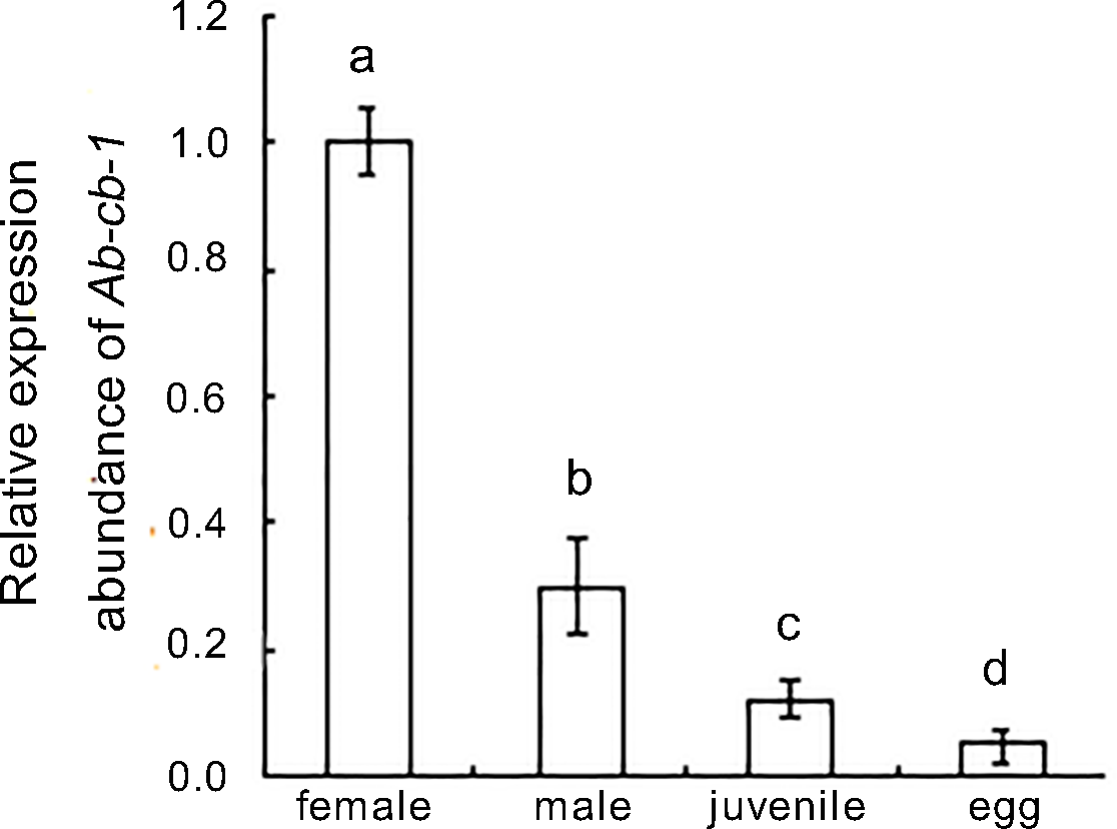
Expression of *Ab-cb-1* in different developmental stages of *Aphelenchoides besseyi*. Bars indicate standard errors of the mean (n = 3), and different letters indicate significant differences (*p*<0.05) between treatments.

*Ab-cb-1* expression levels in the mixed-stage nematodes of two populations and in four developmental stages including eggs, juveniles, females and males of the *Ab*-S24 population were detected by qPCR. The results showed that the relative expression level of *Ab-cb-1* in the *Ab*-S24 population was approximately 6.9 times that in the *Ab-*N10 population, and the difference was significant (*p*<0.05) (Fig 4). The *Ab-cb-1* expression level was highest in females, and the expression levels in males, juveniles and eggs were 30%, 12.2% and 5% of that in females. The differences among the four stages were significant (*p*<0.05) (Fig 5).

### *In situ* hybridization of *Ab-cb*-1

**Fig 6:**
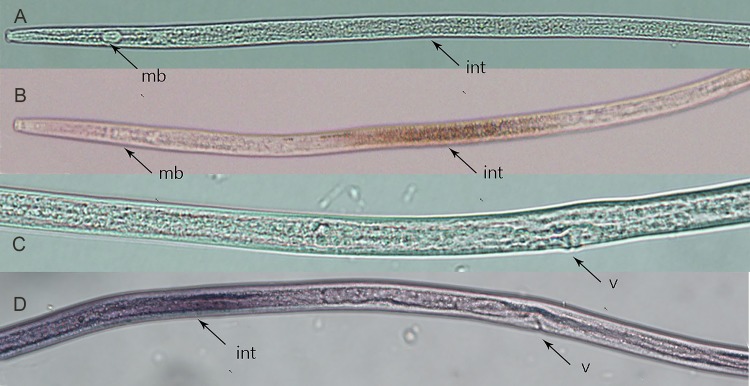
Tissue localization of *Aphelenchoides besseyi* cathepsin B gene *Ab-cb-1* mRNA in females using *in situ* hybridization. A, C: sense *A*. *besseyi cb* DIG-labeled cDNA probe; B, D: antisense *A*. *besseyi cb* DIG-labeled cDNA probe. Images suggested that *Ab*-*cb*-1 was present in the intestine of females. mb: medium bulb; v: vulva; int: intestine.

The results of *in situ* hybridization suggested that *Ab*-*cb*-1 was present in the intestine of females (Fig 6B and 6D). No hybridization signal was detected in nematodes when the control (sense *Ab-cb-1* DIG-labeled RNA) probe was used (Fig 6A and 6C).

### *Ab-cb*-1 RNAi

**Fig 7:**
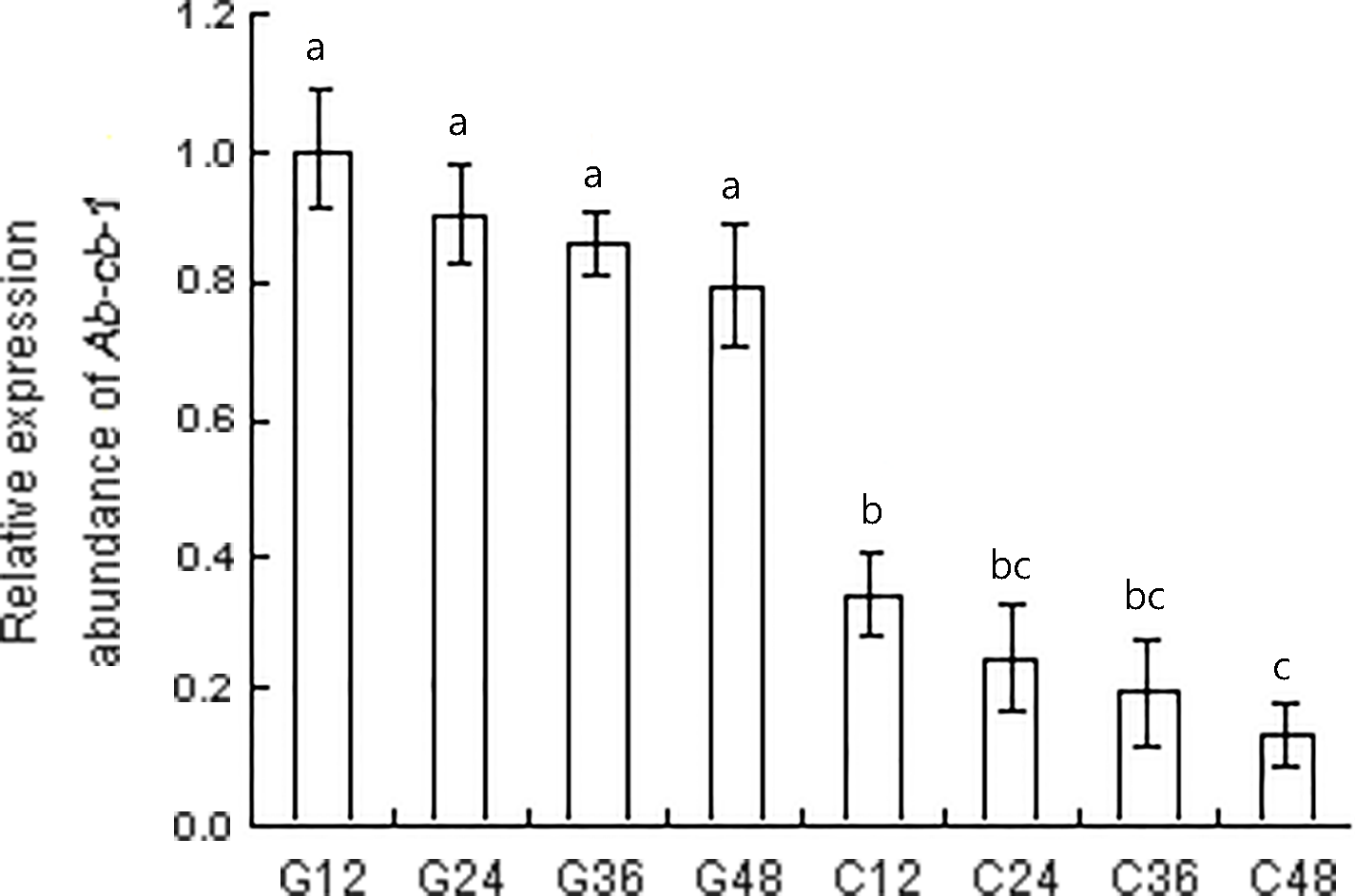
Expression levels of *Ab-cb-1* mRNA in *Aphelenchoides besseyi* treated with *Ab-cb-1* double-stranded RNA (dsRNA). G12, G24, G36 and G48: expression of *Ab-cb-1* mRNA in control nematodes soaked in non-endogenous *gfp* dsRNA solution for 12 h, 24 h, 36 h and 48 h, respectively; C12, C24, C36 and C48: expression of *Ab-cb-1* mRNA in nematodes soaked in *Ab-cb-1* dsRNA for 12 h, 24 h, 36 h and 48 h, respectively. Bars indicate standard errors of the mean (n = 3), and different letters indicate significant differences (*p*<0.05) between treatments.

The RNAi efficiency of *Ab-cb-1* was detected by qPCR after nematodes were treated with *Ab-cb-1* dsRNA. Compared to the expression level of *Ab-cb-1* in nematodes treated with the *gfp* dsRNA, the expression of *Ab-cb-1* in these nematodes decreased significantly, by 65.8%, 65.6%, 66.5% and 66.3% when nematodes were soaked in *Ab-cb-1* dsRNA for 12 h, 24 h, 36 h and 48 h (*p*<0.05). Expression of *Ab-cb-1* decreased with increasing dsRNA treatment time. The expression level of *Ab-cb-1* was the lowest when nematodes were soaked for 48 h; this level was significantly lower than that of nematodes soaked for 12 h (*p*<0.05) but was not significantly different from those of nematodes soaked for 24h and 36 h (*p>*0.05). Nematode *Ab-cb-1* expression was not significantly different among any of the *gfp* dsRNA treatments used as controls in this experiment (*p>*0.05) (Fig 7).

**Fig 8:**
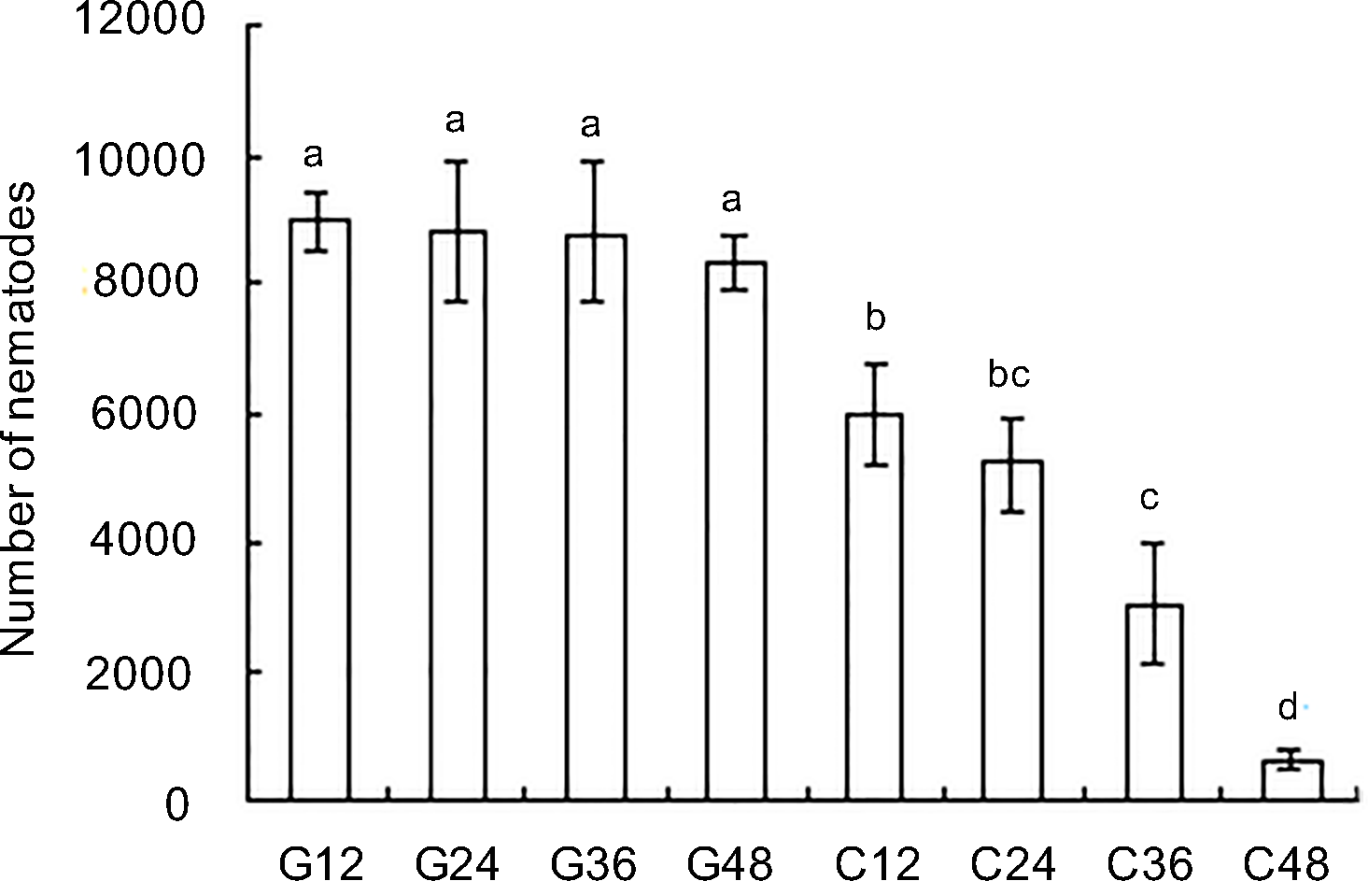
Number of *Aphelenchoides besseyi* on carrot callus after inoculating 30 females for 35days. G12, G24, G36 and G48: the numbers of *A*. *besseyi* after inoculating nematodes treated with non-endogenous *gfp* dsRNA solution for 12 h, 24 h, 36 h and 48 h, respectively; C12, C24, C36 and C48: the numbers of *A*. *besseyi* after inoculating nematodes treated with *Ab-cb-1* dsRNA for 12 h, 24 h, 36 h and 48 h, respectively. Bars indicate standard errors of the mean data (n = 5), and different letters indicate significant differences (*p*<0.05) among treatments.

The effect of *Ab-cb-1* RNAi on reproduction in *A*. *besseyi* was tested by inoculating the nematodes treated with *Ab-cb-1* dsRNA on carrot disks. After culture on carrot disks for 35 d, nematodes treated with *Ab-cb-1* dsRNA for 12 h, 24 h, 36 h and 48 h had significantly lower reproduction than that of *gfp* dsRNA-treated nematodes (*p*<0.05) (Fig 8). In addition, reproduction of the nematodes treated with *Ab-cb-1* dsRNA decreased with increasing treatment time; the differences were significant (*p*<0.05) among different RNAi treatment groups except between 12 h and 24 h and between 24 h and 36 h (*p>*0.05). No significant difference was found among the *gfp* dsRNA treatment groups in this experiment (*p>*0.05). Therefore, *Ab-cb-1* expression in *A*. *besseyi* was effectively inhibited by soaking the nematodes in *Ab-cb-1* dsRNA, and *Ab-cb-1* RNAi depressed the reproduction of *A*. *besseyi*.

## Discussion

In this study, a 1347 bp full-length cDNA of the cathepsin B gene (*Ab-cb-1*) in *A*. *besseyi* was cloned based on an EST fragment that was highly similar to the cathepsin B gene in the cDNA library of *A*. *besseyi*. *Ab-cb-*1 was a differently expressed gene between *A*. *besseyi* populations *Ab-*S24 and *Ab-*N10 and the relative expression level of *Ab-cb-1* in the *Ab*-S24 population was approximately 6.9 times that in the *Ab-*N10 population. The protein *Ab-*CB-1 consisted of 369 amino acids and had a molecular weight of 41.8718 kDa. *Ab-*CB-1 had the highest identity value (64%) to the known amino acid sequence of cathepsin B-like cysteine protease 6 from *Toxocara canis*, and this result was consistent with the results of a phylogenetic analysis. *Ab-*CB-1 contained the cysteine protease catalytic triad of residues (Cys, His, and Asn) and occluding loops. The triad of Cys, His, and Asn residues constitutes an active catalytic domain, which is highly functional and conserved among the cysteine proteases of peptidase family C1, including cathepsin B [20]. The occluding loop is characteristic of only cathepsin B; this loop is responsible for endopeptidase and exopeptidase activity, and its presence seems to indicate that cathepsin B possesses the ability to act [21, 22, 23] as a self-inhibitor by partially blocking the active site when the loop is inactive [24–28]. These structural features indicated that *Ab-*CB-1 had the functions and activities of cathepsin B. *Ab-*CB-1 was inferred to react with substrates directly without protein transportation after synthesis in cells because it did not contain any signal peptide sequence and because the transmembrane signal was weak in the putative transmembrane region.

Cathepsin B (CB) is a member of the cysteine protease family. Cysteine proteases from nematodes have mostly been found to express in gland or intestinal cells and act as the main "digestive enzymes" of nematodes [10], thus playing important roles in the nematode development and invasion process [20, 29, 30, 31]. Cathepsin B was also reported to be related to reproduction: cathepsin B mRNA increased during pregnancy in endometrial epithelia of the porcine uterus and placenta [32]; cathepsin B activity was a useful marker of oocyte quality in bovine oocytes [33]. In this study, we showed that *Ab*-CB-1 could decompose carrot callus, and the gene was expressed in the intestine of *A*. *besseyi*. Therefore, it could be confirmed that *Ab*-CB-1 acted as a digestive enzyme with hydrolytic protease properties and played an important role in the reproduction of *A*. *besseyi*. The method carrot callus tissue for culture of endoparasitic nematodes was established by Reise in 1987 [34]. It was used in several studies that *Aphelenchoides besseyi* can be cultured on carrot callus [1, 35]. *A*. *besseyi* also can be cultured on some fungi, for example, *Botrytis cinerea*. In this study we use carrot callus for nematode cultivation because carrot callus is also a kind of plant material and it is more similar to rice tissue comparing to fungi. And we suppose long time fungi culturing nematodes might lose its pathogenicity to plants.

The reproductivity of *A*. *besseyi* populations *Ab-*S24 and *Ab-*N10 are quite different and the reproductivity of *Ab-*S24 is approximately 19 times that of *Ab-*N10 [16]. Moreover, differences of *Ab-cb-1* expression level were found between two *A*. *besseyi* populations with different reproductive rates. The *Ab-cb-1* expression level of the high reproductive population *Ab-*S24 was significantly higher than that of the low reproductive population *Ab-*N10.Differences in *Ab-cb-1* expression levels were found in the different developmental stages of the *Ab*-S24 population; the highest expression was in females, then males, juveniles and eggs, and this result was consistent with the biological characteristics and functions of the different developmental stages of *A*. *besseyi*. The females of *A*. *besseyi* bear the heaviest reproductive burden and represent the major nematode stage involved in infectivity; also, their bodies are larger than those of other stages, so they need to digest more food to obtain more nutrition and energy. The number and infectivity of the males were lower than those of the females, but compared to the juveniles, males had a well-developed intestine and reproductive functions. As expected, *Ab-cb-1* expression in males was lower than that in females but higher than that in juveniles. Eggs have no functions related to feeding or digestion, and their *Ab-cb-1* expression was the lowest among the four developmental stages. The expression levels of *AC-cathB-1* in the different stages of *Angiostrongy cantonensis* [30] were similar to those of *A*. *besseyi* in this study. However, the expression of *Rs-cb-1* in males of *R*. *similis* was significantly lower than that in other stages. The males of *R*. *similis* do not feed or infect, as the stylet and esophagus have degenerated; their main activity is mating with females. Furthermore, *R*. *similis* can reproduce through parthenogenesis, and the male stage is not necessarily required for the reproduction of the *R*. *similis* species [15].

After treatment with *Ab-cb-1* dsRNA, the expression of *Ab-cb-1* and the reproductivity of *A*. *besseyi* decreased with increasing time. The RNAi efficiency of *Ab-cb-1* was the highest and nematode reproductivity was the lowest when the nematodes were treated with *Ab-cb-1* dsRNA for 48 h. Although the RNAi effect of soaking with dsRNA was not persistent and hereditary, the *Ab-cb-1* RNAi strongly affected the reproduction capacity of the first generation (F_1_) of *A*. *besseyi*. It resulted in a significant decrease in nematodes number of the second generation (F_2_) comparing to control. The life cycle of *A*. *besseyi* was about 6–7 days (25°C) and nematodes could complete approximately 5–6 generations during 35 days cultivation. The difference in nematodes number was started from the F_2_ generation between the number of *A*. *besseyi* in RNAi treatment and the control treatment. Furthermore, it was enlarged after 4–5 generations. This difference was caused by nematodes being soaked with *Ab-cb-1* dsRNA comparing to control treatment. It was reported and demonstrated in many studies that soaking with dsRNA of target gene could affect nematodes in reproduction [1, 14, 15, 36, 37]. Our result agrees with that of a study reported in *R*. *similis* that showed the highest RNAi efficiency when nematodes were treated with *Rs-cb-1* dsRNA for 48 h, and the reproductivity was significantly decreased on carrot callus [15].

## Conclusion

This is the first work to clone a novel cathepsin gene from *A*. *besseyi* and then identify the biological characteristics and functions. These results showed that the cathepsin B gene played an essential role in the reproductivity of nematodes. Overall, the *cb* gene has important research and application value in exploring new methods to control plant nematodes based on biotechnological strategies.

## Supporting information

S1 Fig Putative *Ab*-CB-1 protein sequence. Underscored: a predicted N-glycosylation site; shaded: the occluding loop; italics: the cysteine protease catalytic triad of residues (Cys, His, and Asn); bold: predicted cleavage points of mature and pro-domains; red: glutamine oxyanion hole; box: hemoglobinase motif; double underline: protease S_2_ pocket.
(PDF)
