# Supplementary material for: Molecular identification and functional characterization of the cathepsin B gene (Ab-cb-1) in the plant parasitic nematode Aphelenchoides besseyi
Source: PLoS One. 2018 Jun 29;13(6):e0199935. doi: 10.1371/journal.pone.0199935 (PMC6025850; doi:10.1371/journal.pone.0199935)
Supplement: S1 Fig — Underscored: a predicted N-glycosylation site; shaded: the occluding loop; italics: the cysteine protease catalytic triad of residues (Cys, His, and Asn); bold: predicted cleavage points of mature and pro-domains; red: glutamine oxyanion hole; box: hemoglobinase motif; double underline: protease S2 pocket. (PDF) [file pone.0199935.s001.pdf]

1 MKTKNNDLILDKNNFNHDAYRRMRVSHSAEALEGQELVDYVNGRQNLRTATSNNKFAKYT

61 KEEKKQLMGVLNVRNPIKARRNMSPTRYMDIQ**L**PESFDSRKQWGSMCRSVTEVRDQSSCG

121 S*C*WSFGAVEAMSDRICIHSNGSIQVDLSAKDLLSCCKACGFGCNGGEPFEAWSFWHTSGI

181 VTGSNYTTKKGCQPYTFPECEHHSTKAHFKPCPKELYPTPKCEKTCQEGYDKEYNADKYY

241 GQEPYAVDEDQEAIKKELYLNGPLEVSFEVYDDFLTYSGGVYVHQGGRIDGG*H*AVKLVGW

301 GTENEVPYWIV*AN*SWNTDFGEDGYFRILRGSNECGIEFGVVGGLPDLKRSPHRVHYHHHH

361 SSASSYDIF
